# AI-Mediated Relational Competence in Medical Education: Construct Rationale and Curriculum Maturity Framework

**DOI:** 10.2196/105459

**Published:** 2026-10-27

**Authors:** Robert J McGrath

**Affiliations:** 1 Department of Health Management and Policy, College of Health and Human Services, University of New Hampshire, 4 Library Lane, Durham, NH, 03824, United States, 1 6038625047

**Keywords:** medical education, empathy, trust, competency-based medical education, patient-clinician relationship, curriculum assessment, ambient AI scribe, shared decision-making, AI

## Abstract

AI is quickly becoming a foundational component of many clinical workflows. New tools are continually evolving to enable a host of functionalities, from ambient documentation to enhanced decision support, assistive diagnostic tools, risk prediction, patient triage, and patient communication. As a result, medical education has begun to address issues of AI literacy and the ethics of its use, including some work on competency frameworks, yet comparatively little attention has been given to the relational competencies clinicians require when AI increasingly mediates the patient encounter. Existing AI competency frameworks emphasize technical literacy, evaluation skills, and sometimes ethical awareness, yet the literature suggests that AI tools alter the structure of the patient-clinician relationship itself, raising questions about trust, transparency, empathic presence, and accountable human judgment. The empathy education literature has established that empathic communication is both teachable and assessable, and the AI competency literature has established that clinicians require new knowledge and skills for AI-enabled practice. These 2 bodies of work, however, remain insufficiently integrated. Medical education currently lacks a coherent competency framework for the relational work clinicians must perform when AI tools shape clinical information, communication, documentation, diagnosis, risk prediction, or treatment recommendations. This viewpoint proposes AI-mediated relational competence as a distinct construct for medical education, operationalized through 6 observable subcompetencies: empathic presence, trustworthiness, transparency in AI use, relational judgment, accountable human decision-making, and equity-aware trust-building. A complementary 5-level curriculum maturity model is introduced for evaluating whether medical schools move beyond curricular mention to systematic, competency-based implementation. Together, the construct and maturity framework are intended to provide the conceptual foundation for subsequent empirical work, including online curriculum assessment, national survey research, expert consensus validation, and competency-based assessment instrument development.

## Introduction

### AI-Mediated Care Requires Relational Competence

AI is no longer a peripheral feature of clinical care. As of the US Food and Drug Administration’s June 2026 update of its AI-Enabled Medical Device List, which covers marketing authorizations through March 2026, more than 1500 AI-enabled medical devices had been authorized for use in the United States [1]. Ambient AI scribes, generative language models, and predictive decision-support systems have rapidly entered routine practice, with other reviews documenting AI’s accelerating integration across a breadth of clinical workflows [2-4]. A 2025 evidence synthesis of digital scribes confirmed that these tools have moved from pilot deployments into mainstream documentation workflows at many major health systems within a span of months rather than years [5]. Not surprisingly, the pace of AI integration has quickly outstripped the pace at which medical education curricula have managed to adapt to it.

Responses to AI to date have come primarily through the development of AI literacy and competency frameworks. Scoping reviews and stakeholder analyses have mapped the technical, evaluative, and ethical competencies clinicians require for AI-enabled practice [6,7]. One review identified core AI competency areas to include AI fundamentals, data analysis skills, ethical and legal considerations, and the ability to humanize patient care through effective communication so that the time AI returns to clinicians can be reinvested in compassionate engagement rather than absorbed by other tasks [8].

The pace of curricular uptake has been substantial. Among US medical schools granting Doctors of Medicine and Osteopathic Medicine degrees that responded to the 2024 AAMC (Association of American Medical Colleges) and AACOM (American Association of Colleges of Osteopathic Medicine) Curriculum SCOPE (Structure, Content, Organization, Process, and Evaluation) Survey (182 of 208 schools, 88% response rate), the proportion incorporating AI into their curricula increased from 53% in 2023 to 77% in 2024 [9]. Recent scoping reviews of AI integration across US health professions curricula and of AI in undergraduate clinical-skills education show technical literacy, ethics, clinical applications, and tool use but do not isolate the relational competencies (empathic presence, patient trust, transparency, and accountable judgment) that AI-mediated care requires [10,11]. Additionally, this rapid integration is occurring in the absence of an established competency framework. It is worth noting that the AAMC’s *AI Competencies Across the Learning Continuum* had, by mid-2026, reached a second public draft, with the final report targeted for fall 2026 [12]. Its publicly stated scope centers on foundational AI concepts, the ethical, legal, and professional implications of AI-enabled care, and the capacity to use, evaluate, and collaborate around AI tools across the learning continuum. These are necessary foundations. They are not, however, organized around the relational work of the AI-mediated encounter itself. These include the preservation of empathic presence, the building of patient trust, and the exercise of accountable human judgment when an algorithm is present at the point of care. The construct proposed here is not positioned as an alternative to the emerging national framework but as the relational complement it underweights.

A recurring theme across this literature is that AI competence is not reducible to AI literacy. Clinicians and patients alike express concerns that AI tools may dehumanize the clinical encounter or may threaten shared decision-making, thereby eroding the relational trust that underpins effective care [13-18]. In a widely cited 2018 *Journal of the American Medical Association* (*JAMA*) Viewpoint, Verghese et al [13] warned that AI must always be guided by physicians and that humanism and empathic care must remain the organizing center of clinical work and not be displaced by algorithmic outputs. Others have identified foundational issues such as consent challenges, the diffusion of responsibility to the AI, and underlying latent biases that could permeate algorithmic training data as ongoing barriers to the effective implementation of machine learning in health care [14], although, conceivably, these will continue to improve technologically over time. Others have called for a more refined and nuanced conceptual understanding of trust as it applies to AI in health care, noting that existing frameworks conflate technical reliability with relational trustworthiness [15]. One study of practitioners further noted that many physicians anticipate AI tools will disrupt the therapeutic relationship in ways that are both positive, by creating efficiencies that allow for relationship-building, and negative, by failing to capture the empathic and nonverbal dimensions central to health and mental health care [16].

The central educational question is, therefore, not only whether learners can effectively use AI tools but also whether they can use them in ways that remain empathic, transparent, trustworthy, and accountable.

### Empathy Education Provides a Foundation but Not the Full Answer

Empathy is among the most well-established teachable domains in medical education. A landmark systematic review published in *Academic Medicine* established that empathy can be enhanced through targeted educational interventions such as communication skills training, standardized patient encounters, reflective writing, patient narratives, early clinical exposure, mindfulness practice, and humanities-based teaching more generally [19]. Subsequent systematic reviews have replicated and extended these findings, suggesting that empathy and compassion remain malleable across the undergraduate medical curriculum when interventions are sustained, multimodal, and assessed [20,21]. However, some studies have suggested that medical student empathy can erode during training because of emotional exhaustion and the dissonance between formal teaching and observed practice [22].

While important, much of the foundational empathy literature predates the widespread integration of generative AI in clinical systems. The standardized patient encounters and communication skills curricula on which empathy education has historically relied did not anticipate a clinical environment in which a third agent can both influence and even structure the encounter [13,16,23,24]. A recent patient survey of attitudes toward ambient AI scribes found that patients are generally open to the technology but emphasize that patient education and explicit consent are critical to maintaining trust during AI-mediated encounters [25]. A separate time-motion study showed measurable changes in clinician eye contact and consultation flow when ambient scribes were introduced, underscoring that the dynamics of the encounter itself shift when AI is present [23].

The new educational question is therefore not whether empathy can be taught, but how empathic presence is preserved and expressed during the encounter and how it is assessed when AI tools are in use. Existing empathy frameworks provide a useful foundation, but they require modification to account for the AI-mediated context.

### Trustworthiness Is Distinct From Empathy

Trust is frequently treated as a downstream consequence of empathy in the clinical communication literature, but contemporary work on AI proliferation in health care suggests that trustworthiness ought to be treated as an analytically distinct educational construct. More recently, trust has been defined as a multidimensional concept encompassing competence, reliability, transparency, accountability, fairness, and respect for patient agency, none of which is reducible to empathic communication alone [15]. A second review examined how explainable AI features can both increase and decrease clinicians’ trust, depending on how explanations are framed and whether they support or undermine clinician judgment [26]. London [27] has argued that opaque decisions are more common in medicine than critics often acknowledge and that the demand for algorithmic explainability must be balanced against the moral responsibilities clinicians hold for the decisions they make and explain to patients. Together, these contributions sharpen rather than resolve the educational challenge. Clinicians must learn to communicate in a trustworthy manner even when the underlying algorithmic reasoning is somewhat or wholly opaque.

Patient-facing research reinforces this distinction. A recent *JAMA Network Open* study of US adults reported that overall trust in health systems to use AI responsibly is low, and that this distrust is unevenly distributed across demographic groups, signaling a need for focused organizational trustworthiness and improved communication in addition to empathic care [18]. Patient focus group research further found that patients view AI as a supportive tool but that patients prefer human oversight of automated tools as they are sensitive to the reliability of information delivered by AI and expect their clinician to remain an accountable interpreter of AI [17]. What is unclear, however, is whether these views will continue as AI increasingly pervades patients’ everyday lives.

The clinical implication is that AI-mediated care introduces a new set of questions that empathic communication alone cannot resolve. Patients may reasonably wonder where care recommendations are coming from, be it the clinician, the AI, or perhaps a third-party vendor via algorithmic determinations. They may question how an algorithm trained on a nonrepresentative population applies to them. They also may ask what the clinician would have done in the absence of the AI [14,28-32]. Students therefore need explicit preparation in the trustworthiness and accountable use of AI-supported recommendations, skills that overlap with but are not identical to empathic communication.

## Proposed Construct: AI-Mediated Relational Competence

AI-mediated relational competence is proposed here as the emerging clinician’s ability to preserve empathy and patient trust, improve care transparency and shared decision-making, and exercise equity awareness and accountable human judgment when AI tools have the potential to influence all the elements of the care encounter and the resulting treatment recommendations. The construct integrates existing scholarship on empathy education [19-22], trust in health care AI [14,15,18,26-31], AI competencies for clinicians [6-8,14,33], clinical communication, and competency-based medical education [34-37].

Three features distinguish this construct from existing AI literacy frameworks. First, it is *relational rather than technical*. Where AI literacy frameworks emphasize what the clinician must know about the algorithm, relational competence emphasizes what the clinician must do with that information on behalf of the patient [13,14]. Second, it is *assessable rather than aspirational*. The construct is operationalized through observable learner behaviors that can be taught, practiced, evaluated, and remediated within established competency-based assessments including objective structured clinical examinations (OSCEs), standardized patient encounters, direct observation, and/or portfolio assessment [35,37]. Third, it is *integrative rather than additive*. The construct is not proposed as a new soft skill bolted onto existing curricula. It is a clinical performance domain for technology-enabled practice that maps onto the 8 competency domains synthesized in the Physician Competency Reference Set, in particular, professionalism, interpersonal and communication skills, and patient care [36] domains, and it aligns with the AAMC competency framework currently in development [12].

A recent framework proposed a three-lever approach to concurrent reform of medical AI education, emphasizing that curricular, assessment, and regulatory mechanisms must move together [33]. The construct proposed here is intended to provide the conceptual content that such reform efforts can teach and assess.

The proposed 6 subcompetencies are related but not redundant, and the relationships among them are themselves part of the construct. They can be read along 2 dimensions (Figure 1). The first concerns the relational stance the clinician sustains with the patient, expressed through empathic presence, demonstrated trustworthiness, and equity-aware trust-building. The second concerns the clinician’s conduct toward the AI and its outputs within the encounter, expressed through transparency in AI use, relational judgment, and accountable human decision-making. Relational judgment functions as the hinge between the 2: the capacity to read when the tool is supporting or eroding the relationship and to act accordingly. Because the construct describes relational work, some conceptual overlap among the subcompetencies is expected rather than a defect; the encounter is a single performance, and these are facets of it rather than independent modules.

**Figure 1.**
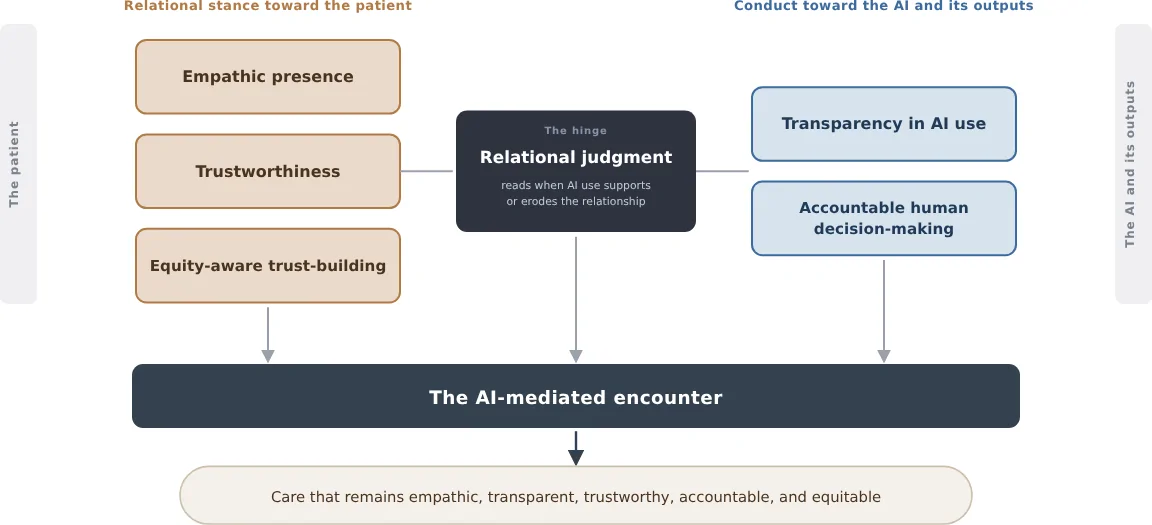
AI-mediated relational competence: a conceptual model of 6 observable subcompetencies across 2 dimensions, bridged by relational judgment.

What justifies treating these facets as distinct assessable targets is that they dissociate in practice. A learner may disclose that AI informed the recommendation (transparency) while uncritically deferring to its output (a failure of accountable decision-making). A learner may communicate warmly and attentively (empathic presence) while overstating the authority of an algorithm (a failure of trustworthiness). Or a learner may explain a tool clearly to one patient while dismissing the well-founded skepticism of another (a failure of equity-aware trust-building). Because these behaviors can be present or absent independently of one another, each can be separately observed, taught, and remediated in a way that a single global rating of “relational skill” would obscure. The construct is therefore neither a relabeling of empathy nor a relabeling of AI ethics. Rather, it specifies the distinct and separately assessable behaviors through which a clinician keeps an AI-mediated encounter empathic, transparent, trustworthy, accountable, and equitable.

## Construct Boundaries and Relationship to Existing Competencies

The relational dimensions of clinical care were well described long before AI entered the encounter. Competency frameworks have consistently named interpersonal and communication skills, professionalism, and patient care as distinct domains of physician performance [36]. Empathy education has established that the capacities underlying those domains are teachable and assessable [19-21], and the competency-based movement has supplied the assessment architecture through which they are evaluated [34,35]. What that literature has not required is a single organizing term. “Relational competence” is used here as an integrative label for capacities that existing frameworks distribute across several domains. It is proposed in this viewpoint rather than adopted from established usage, and the claim advanced is not that these capacities are new but that AI mediation changes the conditions under which they must be enacted and assessed.

The proposed construct is, therefore, best understood as a performance layer rather than a replacement for adjacent domains, each of which answers a different question. AI literacy concerns what the clinician knows about the tool: its inputs, limits, and failure modes. AI ethics concerns the normative questions the tool raises, including fairness, privacy, bias, and the just distribution of benefit and risk. Empathy education concerns the clinician’s recognition of and response to the patient’s experience. Professionalism concerns role fidelity and the standards of conduct to which the clinician is accountable. Calls for a data-informed health workforce sit alongside these as a further technical competency [38]. AI-mediated relational competence names none of these. It names the observable clinical behaviors through which all of them are enacted when AI participates in a specific encounter with a specific patient. Some conceptual overlap with its parent domains is expected, since the encounter is a single performance rather than a set of independent modules.

This raises a reasonable question about whether earlier clinical technologies required the same construct. Electronic health records, rule-based decision support, and telemedicine each altered the encounter, shifting clinician attention toward the screen and generating its own digital-health competency frameworks [39]. Some of these systems incorporated earlier forms of AI well before the recent widespread adoption of generative models. The distinction proposed here is therefore better understood as a matter of degree than of kind, turning on the extent of a system’s participation in the encounter rather than its mere presence. Earlier systems, AI-enabled or not, were principal systems in which the clinician queried a record or a rule set and remained the author of the note and thereby the source of the words the patient heard. Contemporary generative systems are more integrative and proactive. They generate the clinical record rather than store it [40]; they deliver recommendations already formed and carrying implied authority [14,27]; and they can produce patient-facing language that reads as empathic. Each shift relocates something that previously belonged to the clinician. It is that relocation, rather than screen time or workflow disruption, that the construct is designed to address. The technologies principally at issue here are therefore ambient documentation tools, generative language models, and predictive or diagnostic decision-support systems, rather than digital tools generally.

## Observable Subcompetencies

Competency-based medical education requires observable behaviors rather than aspirational values [34,35]. Accordingly, this proposed construct is divided into 6 subcompetencies, each defined in terms of learner behaviors that can be assessed in clinical or simulated settings and evaluated against explicit performance criteria. Table 1 provides the full construct justification matrix. The subcompetencies are empathic presence, trustworthiness, transparency in AI use, relational judgment, accountable human decision-making, and equity-aware trust-building. Curricular maturity is treated separately as an institution-level dimension.

**Table 1.** Construct justification matrix: Subcompetency definition, anchoring, observable learner behaviors, and illustrative survey or curriculum-review questions.

Construct	Definition	Literature anchors	Observable learner behaviors	Survey/curriculum questions
Empathic presence	Capacity to recognize, communicate understanding of, and to respond to a patient’s experience while using digital or AI tools	Empathy is teachable and assessable [19-21,41]; AI shifts the conditions of empathic expression [13,16,23]	Responds to emotion; maintains patient-centered attention; explains screen or AI use; and integrates patient narrative into decisions	Is empathy explicitly taught, practiced, and evaluated in AI-mediated scenarios?
Trustworthiness	Demonstrated capacity to act and communicate in ways that justify patient confidence	Trust is distinct from empathy and multidimensional [15,18,26,28]	Explains clinician role in interpreting AI; acknowledges uncertainty; avoids overstating AI authority; invites patient concern; respects patient agency in decisions; applies consistent standards across patients; and sustains reliability across encounters	Are trust-building and trustworthiness explicit learning objectives, competencies, or assessment domains?
Transparency in AI use	Ability to explain when and how AI informs care in patient-understandable language	AI competency frameworks emphasize safe, ethical use [6-8,14]; patients demand explicit disclosure [17,25]; and explainability remains contested [26,27]	Explains AI-supported recommendations; distinguishes AI input from clinician judgment; and communicates uncertainty and limitations	Do curricula teach disclosure, explanation, and communication of AI-supported decisions?
Relational judgment	Ability to judge when AI use supports or undermines patient-centered care	AI may create distance, confusion, mistrust, overreliance, or inequity [13,16,42]	Recognizes when AI may undermine trust or agency; adapts the encounter; and prioritizes patient concern over tool use	Do students practice AI-mediated cases involving concern, disagreement, bias, or uncertainty?
Accountable human decision-making	Ability to maintain professional responsibility for recommendations shaped by AI	AI may diffuse responsibility [14,26,30]; CBME^a^ should preserve clinician accountability [34-36]	Verifies AI outputs; explains that AI informs but does not replace clinical responsibility; documents reasoning	Do curricula teach accountability for AI-supported recommendations?
Equity-aware trust-building	Ability to recognize how bias, inequity, prior harm, or historical mistrust may shape patient responses to AI-mediated care	Bias and fairness are central to AI ethics [2,14,42] and trust varies across demographic groups [18]	Names bias risks; adapts explanations to patient concerns; avoids dismissing mistrust; and supports shared decision-making	Does AI, trust, and empathy teaching include equity, bias, and vulnerability?
Curricular maturity	Institutional degree to which domains are defined, taught, reinforced, assessed, remediated, and tied to competency expectations	Curricular mention is not competency formation [35,39] and maturity models support empirical assessment	Institution-level construct and not individual learner behavior	Distinguish symbolic inclusion from systematic and competency-based implementation.

a CBME: competency-based medical education.

Empathic presence is the capacity to recognize, communicate understanding of, and respond to a patient’s experience while concurrently using digital or AI tools. It extends established empathy education [19-21] into AI-mediated contexts in which attention and conversational rhythm are altered by the presence of ambient scribes and other screen-mediated workflows [13,16,23]. Validated empathy measurement instruments, such as the Jefferson Scale of Physician Empathy [41], provide the psychometric foundations for assessing empathic orientation in medical learners but were developed prior to widespread AI integration and rely on self-report rather than observed behavior, thus offering a necessary but insufficient starting point for assessing empathic presence in AI-mediated encounters.

A further distinction is necessary as generative systems become capable of producing patient-facing language. Large language models can generate text that reads as warm, attentive, and responsive. Controlled work in medical education has shown that structured practice with role-prompted conversational agents can improve learners’ perceived communication competence across emotionally demanding scenarios [43]. Empathic presence, however, is not reducible to the production of sympathetic phrasing. It requires attention to what is occurring in front of the clinician. This requires sensitivity to context that the model cannot observe and to the professional accountability that attaches to a person rather than a system. AI-generated language may support communication and, in some settings, may improve it, but the clinician remains responsible for interpreting the patient’s situation, judging what the moment requires, and delivering care within a relationship for which someone can be held accountable. Assessment of empathic presence in AI-mediated encounters should therefore target the learner’s attentiveness and judgment rather than the fluency of the language produced.

Trustworthiness is the demonstrated capacity to act and communicate in ways that justify patient confidence. It draws on the expert consensus work, establishing trust as a multidimensional construct distinct from empathy [15], and on patient research, demonstrating that transparency, accountability, and respect for patient agency are central to patient trust in AI-enabled care [17,18,25].

Transparency in AI use is the ability to explain when and how AI informs care in patient-understandable language. This subcompetency reflects both the patient’s need for clear disclosure [17,25] and the clinician’s challenge of explaining AI-supported recommendations where the underlying logic may be opaque [26,27]. Conceptual work on AI-supported shared decision-making argues that effective shared decision-making in AI-enabled care requires clinically meaningful reasoning beyond just technical transparency [44].

Relational judgment is the ability to determine when AI use supports or undermines patient-centered care. AI tools have been shown to create distance between clinicians and patients, encourage an overreliance on AI outputs, and potentially amplify inequity [13,16,42]. Clinical learners must develop the skills to recognize these effects when they are present and then adapt the encounter accordingly.

Accountable human decision-making is the ability to maintain professional responsibility for recommendations shaped by AI. The literature on AI in clinical decision support has frequently identified the risk of responsibility diffusion when algorithmic recommendations enter the workflow [14,26,30]. Competency-based medical education must preserve clinician accountability, requiring learners to verify AI outputs and then convey that AI informs but does not replace clinical judgment. They must then document their reasoning in a way that maintains the clinician as the accountable decision-maker [35,36].

Equity-aware trust-building is the ability to recognize how bias, inequity, prior harm, or historical mistrust may shape patient responses to AI-mediated care. Bias and fairness concerns are central to AI ethics [2,14,42], and these concerns further shape relational legitimacy. Patients from communities with prior experience of medical bias may be more skeptical of algorithmic recommendations and of being distanced from their clinicians, and learners must be prepared to acknowledge and engage with that skepticism rather than dismiss it. The previously cited *JAMA* survey also documented that trust in health systems’ use of AI varies systematically across demographic groups, thus reinforcing the centrality of equity to relational trust-building [18].

Because trustworthiness, transparency in AI use, and accountable human decision-making all concern the clinician’s conduct in relation to the tool, their assessment boundaries warrant statement. Transparency concerns disclosure and whether the patient is told that AI informed their care in terms they can understand and use. Accountable human decision-making concerns verification and ownership and whether the clinician checks the output and retains responsibility for the resulting recommendation. Trustworthiness is broader than either and is assessed at the level of the encounter as a whole, namely whether the clinician’s conduct justifies the patient’s confidence, including respect for patient agency, consistency of care across patients, reliability over time, and candor about the limits of what the tool and the clinician can know. The distinction is observable in practice. A learner may disclose AI use fully and still not be trustworthy, for instance, by disclosing in terms that discourage questions, and a learner may verify an output diligently while conveying a false impression of certainty. Table 2 sets out illustrative competent and concerning behaviors for each subcompetency to support assessment at these boundaries. As with the maturity model and assessment blueprint described here, these behavioral anchors are proposed rather than empirically validated and require confirmation before use in high-stakes assessment or institutional benchmarking.

**Table 2.** Competent and concerning behaviors for each subcompetency.

Subcompetency	Competent behavior	Concerning behavior
Empathic presence	Maintains attention to the patient while using the tool; responds to emotional cues; and explains AI use without disrupting rapport	Fixates on the screen or device; misses or overrides emotional cues; and allows documentation to set the pace of the encounter
Trustworthiness	Acknowledges uncertainty; avoids overstating AI authority; respects patient agency in decisions; applies consistent standards across patients; and sustains reliability across encounters	Presents AI output as definitive or unquestionable; dismisses patient preferences; and varies candor according to the patient
Transparency in AI use	Explains in patient-understandable language when and how AI informed care and distinguishes AI input from clinician judgment	Conceals AI involvement; relies on technical jargon that forecloses questions; and discloses in terms the patient cannot act on
Relational judgment	Recognizes when AI use is undermining comfort, agency, or disclosure; pauses or adapts tool use accordingly	Continues tool use despite visible patient discomfort and prioritizes workflow over the patient’s evident concern
Accountable human decision-making	Verifies AI output against clinical findings; frames the recommendation as clinical judgment; and documents reasoning	Defers to the algorithm without examination; attributes the decision to the system; and cannot articulate a rationale independent of the tool
Equity-aware trust-building	Validates concerns about bias; explains known limitations and safeguards; and adapts the explanation to the patient’s stated concern	Dismisses mistrust as irrational or uninformed; applies a uniform explanation regardless of concern; and treats skepticism as nonadherence

Three clinical vignettes illustrating the construct in practice, offered as proposed rather than empirically validated teaching examples, are provided in Multimedia Appendix 1: an ambient documentation tool that is running when a patient hesitates to disclose that cost has prevented them from taking a medication as prescribed; a patient who asks whether an AI-generated low-risk assessment reflects the tool’s judgment or the clinician’s; and a patient who asks whether a risk model was developed using data from patients like them. Each vignette identifies the subcompetencies it engages, contrasts a competent response with a plausible failure, and indicates where the scenario could be assessed.

Each subcompetency defined here can further be mapped to established teaching and assessment modalities [24,35,37]. Recent work has demonstrated the feasibility of AI-simulated patient interactions for training and assessing clinical interview skills [24], suggesting that AI-supported assessment infrastructure is actively developing in parallel with the educational challenges that AI itself creates.

## Curricular Maturity

The key empirical question raised by this construct is not whether medical schools *mention* trust, empathy, or even AI in their curricular outlines. Nearly all do in some form, whether in mission language, course and syllabus catalogs, or in stand-alone AI literacy sessions [7]. The more consequential question is whether schools *systematically* teach, reinforce, and assess these competencies.

This distinction is well-recognized in competency-based medical education scholarship. The transition from time-based to outcomes-based training has highlighted persistent gaps between what curricula declare and what learners can demonstrably do [34,35,37]. A recent study on the design and implementation of a digital competence framework for health professionals emphasized that competence assessment must be tied to defined frameworks and tested in real professional contexts rather than inferred from curricular inclusion [39]. Table 3 presents a 5-level curriculum maturity model for AI-mediated relational competence, ranging from *implicit or absent* (level 1) through *introduced*, *integrated*, and *assessed* to *competency-based* (level 5), with the latter defined by linkage to feedback, remediation, progression, and entrustment decisions.

**Table 3.** Curriculum maturity levels for AI-mediated relational competence: proposed level, label, and definitions.

Level	Label	Description
1	Implicit or absent	Trust, empathy, and AI-mediated relational competence are assumed, modeled informally, or not visible in curriculum documentation.
2	Introduced	Topics are named in isolated sessions, learning objectives, or professionalism language, without longitudinal reinforcement.
3	Integrated	Topics are reinforced across multiple courses, clerkships, phases, or required learning experiences.
4	Assessed	Learners are evaluated using rubrics, OSCEs^a^, standardized patients, direct observation, portfolios, or patient feedback.
5	Competency-based	Performance is linked to feedback, remediation, progression, graduation expectations, or entrustment decisions.

a OSCEs: Objective Structured Clinical Examinations.

The maturity model is proposed rather than validated. Its levels are derived from established distinctions in competency-based medical education between curricular mention, longitudinal reinforcement, formal assessment, and entrustment [34,35], but they have not yet been subjected to consensus or psychometric evaluation, and they should not be used for high-stakes curriculum evaluation or institutional comparison on the strength of this viewpoint alone. A validation program would proceed in stages: expert consensus review to confirm that the levels are exhaustive, mutually exclusive, and correctly ordered; cognitive interviewing with curriculum leaders to establish that the level descriptors are interpreted consistently across institutions; pilot coding of publicly available curriculum documents with interrater reliability testing to determine whether independent coders assign the same level to the same program; and systematic collection of validity evidence linking assigned levels to observable learner performance [37]. Until that evidence exists, the model is best used formatively as a shared vocabulary for institutional self-assessment and curricular planning rather than as a benchmarking instrument.

This framework is intended to serve 2 empirical purposes. First, it can support online curriculum assessment, in which publicly available curriculum materials are coded against the maturity levels to produce a national baseline picture of where US medical schools currently stand. Second, it can inform more systematic national survey research of medical and clinical programs. Comparing what schools publicly document with what curriculum leaders report is itself a useful empirical question.

## Implications for Survey Development and Curriculum Assessment

A construct framework is a methodological precondition for both online curriculum assessment and national survey research. Without an explicit construct, coders cannot reliably distinguish symbolic from systematic inclusion, and survey items cannot be mapped to a coherent underlying competency model. The framework proposed here is designed to support both.

For online curriculum assessment, the construct provides a coding rubric that can be applied to publicly available materials and to the AAMC Curriculum Inventory. Each subcompetency from Table 1 generates a set of indicators (keywords, learning objectives, assessment modalities, and course types) that coders can search for and classify against the maturity levels of Table 3. This methodology mirrors published approaches to curricular content analysis in medical education.

For national survey development, the construct provides item domains corresponding to each subcompetency and maturity level. Items can ask not only whether a topic is taught but also whether it is reinforced longitudinally, assessed with explicit criteria, reflected upon for remediation, and linked to progression decisions.

Table 4 sets out an illustrative assessment blueprint, indicating how the subcompetencies might be distributed across settings and methods already available in most programs, from preclinical standardized patient encounters through workplace-based observation, simulation, and portfolio reflection. The blueprint is a worked example rather than a validated instrument. It has not been piloted; the mapping of subcompetencies to settings is one defensible arrangement among several; and the validation requirements stated for the maturity model apply to it equally. Its purpose is to show that the construct can be assessed using infrastructure that programs already operate, rather than to prescribe how any particular program should do so.

**Table 4.** Illustrative assessment blueprint for AI-mediated relational competence.

Setting and scenario	Subcompetencies assessed	Assessment method
Preclinical OSCE^a^: the learner introduces an ambient documentation tool at the start of a sensitive history.	Empathic presence; transparency in AI use; and relational judgment	Standardized patient checklist with faculty global rating
Clerkship workplace-based observation: the learner discusses an AI-generated risk estimate with a patient who questions it.	Trustworthiness and accountable human decision-making	Direct observation with narrative feedback
Simulation: an AI tool produces a biased or incorrect recommendation that the learner must recognize and address.	Relational judgment; equity-aware trust-building; and accountable human decision-making	Structured debrief with rubric-based rating
Portfolio: the learner reflects on an encounter in which a patient expressed mistrust of AI-supported care.	Equity-aware trust-building and trustworthiness	Reflection rubric with faculty feedback

a OSCE: Objective Structured Clinical Examinations.

The competent and concerning behaviors in Table 2 correspond to the 6 subcompetencies defined above and are intended to complement, rather than duplicate, the maturity levels in Table 3. Table 3 describes how deeply a curriculum addresses the construct, while Table 2 describes what competent and concerning performance looks like within any given encounter.

Table 4 builds on both preceding tables by mapping the same subcompetencies onto concrete assessment settings and methods already available in most training programs.

## Faculty Development and the Hidden Curriculum

Curricular maturity describes what an institution teaches. It does not describe what learners observe. The values and behaviors transmitted through routine clinical practice, rather than through formal instruction, are recognized influences on professional formation and plausible contributors to the erosion of empathy documented during training [22]. AI-mediated relational competence is exposed to the same dynamic. A learner taught to disclose AI use, verify AI outputs, and address patients’ concerns about algorithmic fairness will discount that teaching if supervising clinicians do not follow suit and reinforce these behaviors.

Faculty development is therefore a condition of the construct’s viability rather than an implementation detail. Supervising clinicians require preparation to model the behaviors specified in Table 1 by disclosing AI use in patient-understandable terms, seeking patient agreement where documentation tools are in use, verifying AI outputs before acting on them, recognizing automation bias in their own reasoning, and engaging concerns about fairness and reliability with the seriousness afforded other clinical questions. Evidence from AI-generated educational documentation is instructive here. Mischaracterizations and hallucinations occurred at low but nontrivial rates and were largely corrected when faculty reviewed the output before submission [40]. The findings illustrate the potential value and feasibility of faculty verification in that setting. An institution could rate highly on formal curricular provision while its clinical environment models the opposite, and self-assessment against Table 3 should be interpreted with that limitation in view.

## Conclusion

AI does not render trust and empathy obsolete in medical education; rather, it raises the importance of both as foundational human inputs to the care encounter. As AI tools enter routine clinical workflows, the relational skills clinicians perform with patients become more challenging and also more critical to assess. The existing AI competency literature has identified the technical, evaluative, and ethical skills clinicians need [6-8,14,33], and the existing empathy education literature has similarly established that relational skills can be taught and assessed [19-22,41]. What has been missing is a competency framework that bridges these 2 bodies of work and operationalizes the relational competencies required for AI-mediated practice.

The construct proposed here and the accompanying curriculum maturity model are offered as the bridge. The framework shifts the question from *whether* trust and empathy are mentioned in medical curricula to *whether* they are taught, practiced, assessed, and required as observable performance in AI-enabled care. The rationale for this construct is intended to provide a conceptual foundation for subsequent empirical work.

## Supplementary material

10.2196/105459 Multimedia Appendix 1 Clinical vignettes illustrating AI-mediated relational competence.

## Abbreviations

AACOM: American Association of Colleges of Osteopathic Medicine
AAMC: Association of American Medical Colleges
*JAMA*: *Journal of the American Medical Association*
OSCE: objective structured clinical examination
SCOPE: Structure, Content, Organization, Process, and Evaluation
