# Supplementary material for: AI-Mediated Relational Competence in Medical Education: Construct Rationale and Curriculum Maturity Framework
Source: JMIR Med Educ. 2026 Oct 27;12:e105459. doi: 10.2196/105459 (PMC13717324; doi:10.2196/105459)
Supplement: Supplementary file 1 [file mededu-v12-e105459-s001.docx]

**Multimedia Appendix 1. Clinical Vignettes Illustrating AI-Mediated Relational Competence**

These three vignettes illustrate how the six sub-competencies operate in practice. Each is composite and illustrative rather than drawn from a specific patient encounter. For each, a competent response is described, the sub-competencies principally engaged are identified, a contrasting response is given to show how the same encounter can fail, and a note indicates where the scenario could be assessed. The competent and concerning behaviors referenced correspond to those specified in Table 3 of the main manuscript.

# **Vignette 1. Ambient documentation during a sensitive disclosure**

**Scenario.** A third-year student is conducting a follow-up visit for type 2 diabetes. An ambient documentation tool is running, routine in the clinic, and the patient consented to its use at check-in. Asked about adherence, the patient says they have been cutting tablets in half to make the prescription last because they cannot afford the refills. They then stop mid-sentence, glance at the device on the desk, and ask who reads these notes.

**Competent response.** The student treats the question as the substantive concern it is rather than as an interruption to the history. They explain that the device is transcribing the conversation and ask whether the patient would prefer it be turned off. They stop the recording and state plainly what has been captured so far. They explain what will and will not appear in the visit note, and, generally, who has access to that note. They then invite the patient to continue, and afterward document the adherence problem themselves, in terms the patient has agreed to, so that the cost barrier is recorded in a way that supports the patient's care rather than exposing more than the patient intended.

**Sub-competencies engaged.** Empathic presence in attending to the patient's hesitation rather than pressing on with the history. Relational judgment in recognizing that a tool, useful moments earlier, had become an obstacle to disclosure. Transparency in AI use in explaining the tool's reach in terms the patient can act on. Trustworthiness in restoring the patient's control over what is recorded.

**Contrasting response.** The student answers briefly that the note is confidential and continues the history or silently stops the recording without explaining what has already been captured. In the second case the tool is switched off but the patient is left uncertain what exists in the record, which addresses the device without addressing the concern. Left unattended are the other sub competencies and patient self-advocacy.

**Assessment note.** Suited to a preclinical and objectively structured clinical examination. The discriminating behavior is whether the learner acts on the patient's hesitation without being prompted, and whether the explanation offered is specific enough for the patient to know what happens to the disclosure.

# **Vignette 2. A patient questions an AI-generated risk estimate**

**Scenario.** A patient in their fifties presents to the emergency department with chest pain. A decision-support tool integrated into the record classifies them as low risk and suggests discharge with outpatient follow-up. When the resident explains the plan, the patient asks whether that is the computer's opinion or the resident's.

**Competent response.** The resident treats the question as reasonable rather than as resistance. They explain what the tool takes into account and what it does not and distinguishes its output from their own assessment. They explain that the tool weighs certain variables, and separately they have examined the patient, reviewed the tracing and the troponin, and formed a view. They state the plan as their own recommendation, what considerations or conditions would warrant changes to that plan or patient actions, and give explicit return precautions. They document their clinical reasoning rather than the tool's output alone.

**Sub-competencies engaged.** Transparency in AI use in distinguishing algorithmic input from clinical judgment. Accountable human decision-making in owning the recommendation and documenting an independent rationale. Trustworthiness in conveying appropriate rather than overstated certainty.

**Contrasting response.** The resident answers that the tool is highly accurate and has been validated on large populations. The statement may be true yet tis still constitutes a failure of accountable decision-making, because it transfers the basis of the recommendation to the system and leaves the patient with no clinician-held rationale to weigh.

**Assessment note.** Suited to workplace-based observation during a clerkship. The discriminating behavior is whether the learner can articulate a rationale that stands independently of the tool's output.

# **Vignette 3. A patient asks whether the tool applies to them**

**Scenario.** A patient in their sixties with poorly controlled hypertension is offered a treatment intensification supported by an AI-derived risk model. They say they have read that such tools do not always perform equally well for everyone and ask whether the model was developed using data from patients like them.

**Competent response.** The resident treats the question as a question about evidence, which is what it is. They say what they know about the model's development population and state plainly where their knowledge ends, rather than offering reassurance they cannot support. They explain what the institution does to monitor performance across groups and note that the recommendation is consistent with their own clinical assessment and with guideline-based care independent of the model. They invite the patient to decide how to proceed given that information and treat the patient's hesitation as reasonable.

**Sub-competencies engaged.** Equity-aware trust-building in recognizing the concern as well-founded rather than as an obstacle. Transparency in AI use in being explicit about the limits of what is known. Trustworthiness in declining to overstate or over-assure. Accountable human decision-making in offering a clinical rationale that does not depend on the model.

**Contrasting response.** The resident offers general reassurance that the tool is well validated or acknowledges the concern but then proceeds as though it had not been raised. Either response treats a question about the evidence as a matter of persuasion, and suggests to observing learners that patient skepticism about AI is something to be managed. A further failure would be to treat this question as characteristic of particular patients rather than as a reasonable question about evidence. The competence lies in being able to answer it for any patient who asks.

**Assessment note.** Suited to simulation with structured debriefing, or to portfolio reflection. The discriminating behavior is whether the learner can say that something is not known and sustain the patient's trust while doing so.
